# Animal Models of Childhood Exposure to Lead or Manganese: Evidence for Impaired Attention, Impulse Control, and Affect Regulation and Assessment of Potential Therapies

**DOI:** 10.1007/s13311-023-01345-9

**Published:** 2023-02-28

**Authors:** Donald R. Smith, Barbara J. Strupp

**Affiliations:** 1 Department of Microbiology and Environmental Toxicology, University of California, Santa Cruz, CA 95060 USA; 2 Division of Nutritional Sciences and Department of Psychology, Cornell University, Ithaca, NY 14853 USA

**Keywords:** Attention deficit, ADHD, Neurotoxicity, Lead, Manganese, Animal model

## Abstract

Behavioral disorders involving attention and impulse control dysfunction, such as ADHD, are among the most prevalent disorders in children and adolescents, with significant impact on their lives. The etiology of these disorders is not well understood, but is recognized to be multifactorial, with studies reporting associations with polygenic and environmental risk factors, including toxicant exposure. Environmental epidemiological studies, while good at establishing associations with a variety of environmental and genetic risk factors, cannot establish causality. Animal models of behavioral disorders, when properly designed, can play an essential role in establishing causal relationships between environmental risk factors and a disorder, as well as provide model systems for elucidating underlying neural mechanisms and testing therapies. Here, we review how animal model studies of developmental lead or manganese exposure have been pivotal in (1) establishing a causal relationship between developmental exposure and lasting dysfunction in the domains of attention, impulse control, and affect regulation, and (2) testing the efficacy of specific therapeutic approaches for alleviating the lasting deficits. The lead and manganese case studies illustrate how animal models can advance knowledge in ways that are not possible in human studies. For example, in contrast to the Treatment of Lead Poisoned Children (TLC) human clinical trial evaluating succimer chelation efficacy to improve cognitive functioning in lead-exposed children, our developmental lead exposure animal model showed that succimer chelation can produce lasting cognitive benefits if chelation sufficiently reduces brain lead levels. In addition, this study revealed that succimer treatment in the absence of lead exposure produces lasting cognitive dysfunction, highlighting potential risks of chelation in off-label uses, such as the treatment of autistic children without a history of lead exposure. Our animal model of developmental manganese exposure has demonstrated that manganese can cause lasting attentional and sensorimotor deficits, akin to an ADHD-inattentive behavioral phenotype, thereby providing insights into the role of environmental exposures as contributors to ADHD. These studies have also shown that oral methylphenidate (Ritalin) can fully alleviate the deficits produced by early developmental Mn exposure. Future work should continue to focus on the development and use of animal models that appropriately recapitulate the complex behavioral phenotypes of behavioral disorders, in order to determine the mechanistic basis for the behavioral deficits caused by developmental exposure to environmental toxicants, and the efficacy of existing and emerging therapies.

**Supplementary Information:**

The online version contains supplementary material available at 10.1007/s13311-023-01345-9.

## Introduction

Behavioral disorders, particularly those involving learning, attention, and impulse control, are among the most prevalent disorders in children [1]. While the etiologies of behavioral disorders are often poorly understood, they are generally recognized to be multifactorial in nature, with epidemiological studies providing evidence for both genetic and environmental risk factors. For example, substantial evidence has emerged over the past several decades supporting associations between various environmental factors and increased risk of behavioral disorders, such as attention-deficit hyperactivity disorder (ADHD) and autism spectrum disorders (ASD) in children and adolescents [1–8]. These environmental risk factors include toxicant exposure, which may vary in magnitude, duration, and life stage, and interact with genetic predisposition.

The reported associations between environmental exposures and increased risk for behavioral disorders have raised significant public health concerns, in part because of the profound disability produced by these disorders, but also because infants are especially vulnerable to environmental toxicants due to susceptible neurodevelopmental processes unique to early life stages [9–12]. For example, the prefrontal cortex (PFC) is uniquely vulnerable to early life insults, often manifesting as impairments in attention and impulse control [13–17]. These reports underscore the need to understand how developmental exposure to environmental toxicants impacts neurobehavioral function and contribute to neuropsychiatric disorders, in order to devise effective intervention and treatment strategies [18, 19].

While epidemiologic studies have played key roles in highlighting potential associations between neurotoxicant exposure and increased risk of various behavioral disorders, they are unable to establish causality, in large part because of challenges with accurate exposure assessment, and because contaminant exposures often occur within the context of sociodemographic factors that themselves place children at risk for impaired cognitive and emotional development (e.g., poverty, poor health care, low maternal education and IQ, maternal depression, low intellectual stimulation in the home). Thus, reported associations between the exposure and increased risk of these disorders may be spurious, reflecting instead the influence of some other confounded risk factor.

Given this, the demonstration of behavioral dysfunction in animal models, under conditions in which confounded risk factors are absent and the exposure is randomly assigned, is essential in establishing causal relationships between the environmental factor (e.g., contaminant exposure) and the disorder, and in elucidating underlying neural mechanisms [16, 17, 20–24]. Moreover, there is a significant body of evidence demonstrating that animal model findings can be readily translatable to human and clinical conditions, provided the animal study design sufficiently recapitulates exposure scenarios and behavioral endpoints relevant to humans (discussed in [20]). Further support for the validity of using rodent and non-human primate models to study the neurobehavioral effects of toxicant exposure is provided by studies demonstrating correspondence in the functional roles of various neural systems between rats, monkeys, and humans. Such correspondence is very clear for the functional effects of damage to structures such as the amygdala [25], hippocampus [26–28], basal ganglia [29], and the frontal cortex [27, 30–32].

A focus of our work, and an emphasis in this review, is the development of animal models using behavioral tests that recapitulate the various phenotypic features of behavioral disorders in children, including impairments in attention, impulse control, and arousal regulation. In particular, our focus has been to elucidate the role that the neurotoxicants lead (Pb) and manganese (Mn) play in syndromes characterized by these areas of dysfunction. This emphasis is well-justified for several reasons. First, attention and impulse control dysfunction, including ADHD, characterize the most prevalent neurodevelopmental syndrome among children, affecting ~ 5–7% of youths up through age 18 years, and ~ 2–3% of adults [1, 33–38]. Second, ADHD and related syndromes are associated with substantial economic costs to patients, families, and society as a whole [1]. A final compelling need to better understand the role of environmental neurotoxicants in the etiology of ADHD and related symptoms is that this type of disorder appears to disproportionately affect young people of color [39], a demographic that often also suffers significant socioeconomic, nutritional, and health disparities, as well as environments with higher pollution burdens compared to the general population [40–43].

## Epidemiology of Multifactorial Attention, Impulse Control, and Affect Regulation Disorders Associated with Environmental Risk Factors in Children and Adolescents

There are three different clinically recognized phenotypes of ADHD: (1) predominantly inattentive, (2) predominantly hyperactive/impulsive, and (3) combined inattentive and hyperactive/impulsive [44]. Genome-wide association studies indicate that numerous common genetic variants account for ~ 40% of the heritability of ADHD [45], and candidate gene studies have identified dopamine, noradrenaline, serotonin, and neurite outgrowth systems as being associated with ADHD [4, 46–51]. In addition, environmental factors, including environmental chemical exposure, are also associated with ADHD risk. These environmental exposure risk factors include pre- or early postnatal exposure to neurotoxicants such as cigarette smoke, alcohol, and air pollution, as well as lead, arsenic, manganese, methylmercury, polychlorinated biphenyls, toluene, chlorpyrifos, dichlorodiphenyltrichloroethane, tetrachloroethylene, and the polybrominated diphenyl ethers, to name a few [1, 5–7, 24, 37, 52–61]. Overall, it has been posited that the combination of multiple environmental and biological risk factors, as is often the case, likely surpasses a certain ADHD threshold, leading to the development of impairing ADHD symptoms [62].

## Environmental Exposure to Lead or Manganese Is Associated with ADHD and Related Attention/Impulse Control Symptoms

Epidemiological and experimental animal model studies, when properly designed, are highly complementary regarding the insights they provide into whether and how environmental exposures may lead to adverse health outcomes, including behavioral disorders like ADHD. As noted above, in this review, we focus on our work concerning animal models of environmental exposure to lead or manganese, specifically as it relates to their roles in producing lasting impairments in attention, impulse control, and arousal regulation. In addition, we discuss the use of these animal models to test the efficacy of potential therapies to alleviate the impairments. In our animal model of developmental lead exposure, we tested the efficacy of chelation therapy with succimer, the most widely used lead-chelating agent in the treatment of pediatric lead poisoning. For the animal model of developmental manganese exposure, we focus on the efficacy of methylphenidate (Ritalin), the most widely prescribed drug for treating ADHD in children. Finally, we discuss how findings from these animal model studies have informed the specific causes of a syndrome characterized by attention and impulse control problems, which is believed to have multiple causes in children, but where the cause(s) in any given case is generally unknown.

It is beyond the scope of this review to comprehensively discuss the existing literature on animal models of developmental exposure to lead or manganese, as a means to place our work into the context of the broader literature. However, we should note that although numerous animal model studies have demonstrated lasting behavioral effects of developmental exposure to each of these neurotoxicants (e.g., [21, 23, 63–67]), very few have included endpoints specific to attentional function or inhibitory control (reviewed in [68–71]), and none of these tested potential therapies related to these specific areas of dysfunction.

## Public Health Threat to Neurobehavioral Function Posed by Environmental Lead Exposure

Lead is among the most well-studied anthropogenic contaminant, with recognition of its neurotoxic potential emerging centuries, if not millennia, ago [72–75]. Environmental lead exposure continues to pose a significant public health problem globally due to large-scale contamination of the biosphere, producing elevated lead levels in contemporary humans that are > 50–500 times greater than in our pre-industrial ancestors [76–78]. Environmental lead exposure during early development occurs primarily through ingestion or inhalation of lead-contaminated media (dietary items, water, dust, etc.). Numerous epidemiological and animal model studies have provided evidence that lead exposure produces lasting cognitive deficits, even at low exposure levels not associated with overt toxicity [16, 78–85]. Moreover, the Centers for Disease Control and Prevention's Advisory Committee on Childhood Lead Poisoning Prevention concluded that there is no level of lead in children that is without deleterious effects [86]. This underscores the significant concerns over the prevalence of low-level lead poisoning in children, given that a recent review by the American Academy of Pediatrics reported that ~ 2.6% of preschool children in the USA had an elevated blood lead concentration ≥ 5 μg/dL [78].

## Epidemiological Evidence Linking Environmental Lead Exposure to ADHD

Whereas there are numerous epidemiological studies reporting associations between elevated lead exposure and adverse cognitive and neurological outcomes in children [78–82], relatively few have systematically evaluated associations between lead exposure and attention/impulse control symptoms, including ADHD [1, 87]. Several cross-sectional studies using data from the National Health and Nutrition Examination Survey in the USA reported that children with elevated blood lead levels were ~ 2–4 times more likely to have been diagnosed with ADHD compared with children with lower blood lead levels [59, 88]. More recently, studies have reported correlations between body lead burden and inattention and/or hyperactivity-impulsivity symptoms [89], and in the case of a relatively recent meta-analysis, higher blood lead levels were associated with a nearly four-fold increase in the odds ratio of ADHD [90]. Finally, Hong et al. [91] reported that, when adjusted for demographic characteristics and other environmental exposures, etc., a ten-fold increase in blood lead concentration was associated with lower Full-Scale IQ and higher parent- and teacher-rated hyperactivity/impulsivity scores and commission errors. 

## Therapeutic Approaches for Treating Neurobehavioral Effects of Lead Exposure

Treatment with lead-chelating agents has been the primary mode of therapy for treating lead-poisoned children since the 1950s, but the metric for gauging therapeutic efficacy has changed over time. When lead-chelating agents were first implemented for clinical use, they were often deployed in the treatment of symptomatic children, some exhibiting signs of lead-induced encephalopathy. The chelating agents used at that time, including CaNa_2_EDTA, are credited with dramatically reducing the mortality rate in such children [92]. Fortunately, blood lead levels of children have declined dramatically over the past 50 + years (though exposure disparities remain) [42, 43, 78], and lead-induced encephalopathy is rare, particularly in developed countries. However, in light of the evidence that even slightly elevated lead levels are associated with impaired cognitive functioning in children [78–80, 82, 86], there remains pressure for clinicians to prescribe chelation therapy at low exposure (blood lead) levels.

While a primary goal of chelation therapy in treating lead-poisoned children is simply to reduce body lead burden, especially in sensitive target organs like the brain, there is also the expectation that reduced brain lead levels will reduce neurotoxicity and associated symptoms. However, surprisingly few studies have evaluated whether chelation therapy improves cognitive outcomes in children with sub-clinical lead poisoning. With respect to succimer, the most widely administered chelating agent for the treatment of lead-poisoned children, only one clinical trial conducted to date included cognitive outcomes [93, 94]. This study, referred to as the Treatment of Lead-Exposed Children (TLC) study, did not detect a benefit of chelation, relative to placebo, in children with blood lead levels between 20 and 44 µg/dL, treated at 12 to 33 months of age. Cognitive benefits from chelation were not seen at the 36-month follow-up [93] or at age 7 years [94]. Notably, these investigators included an assessment of intellectual attainment [the Wechsler Preschool and Primary Scales of Intelligence–Revised (WPPSI-R)], as well as a neuropsychological test battery [the Developmental neuropsychological assessment (NEPSY)] designed to identify neuropsychological deficits that interfere with learning, including attentional and executive functions. Also included were several parent and teacher rating scales designed to tap ADHD as well as behavioral conduct problems [the short form of the Conners’ Parent Rating Scale–Revised (CPRS-R), and the Behavioral assessment system for children-parent rating scale (BASC) for parents and for teachers]. In contrast to the negative findings seen with chelation in this multi-center human clinical trial, a few animal studies suggested that succimer chelation therapy can improve behavioral outcomes caused by lead poisoning. In addition to our studies with numerous cognitive endpoints ([16, 83], discussed below), several animal model studies reported that succimer normalized various behaviors altered by lead exposure, including forced-swim immobility [95], activity level, and habituation rate [96].

## Animal Model of Developmental Lead Exposure, Attention and Impulse Control Impairment, and Therapeutic Efficacy of Succimer Chelation

Our lab has conducted numerous lead exposure studies with various exposure models, with the goal of specifying the nature of the cognitive and/or affective changes produced by early asymptomatic lead exposure, and providing a model system for studies designed to test potential therapies [16, 83, 85, 97–102]. Due to space constraints, we will focus on one large study that tested the effectiveness of the chelating agent succimer to ameliorate lead-induced cognitive deficits [16]. This study, which was designed to parallel the Treatment of Lead Poisoned Children (TLC) multi-center placebo-controlled study on succimer efficacy to improve cognitive function in children [93, 94], sought to determine whether treatment with a succimer regimen shown to produce significant reductions in blood and brain lead levels [97, 102, 103] also lessens the lasting cognitive and affective changes that are produced by developmental lead exposure. It also aimed to determine whether succimer produces lasting cognitive and/or affective impairment when administered in the absence of lead exposure. Findings from the latter are important to gauge the safety of prolonged chelation regimens when treating lead-exposed children, as well as the safety of the drug in off-label uses with no evidence of lead poisoning (e.g., for treating autism), as advocated on numerous websites and organizations such as the American College for Advancement in Medicine. Overall, this study sheds light on the nature of the cognitive and affective changes produced by early lead exposure, provides one of the first tests of the efficacy of succimer chelation on cognitive outcomes in a rodent model, and provides the first evidence for lasting adverse effects of succimer chelation in a common off-label use of the therapy, namely for treating autism [16, 83].

### Study Design

**Fig. 1:**
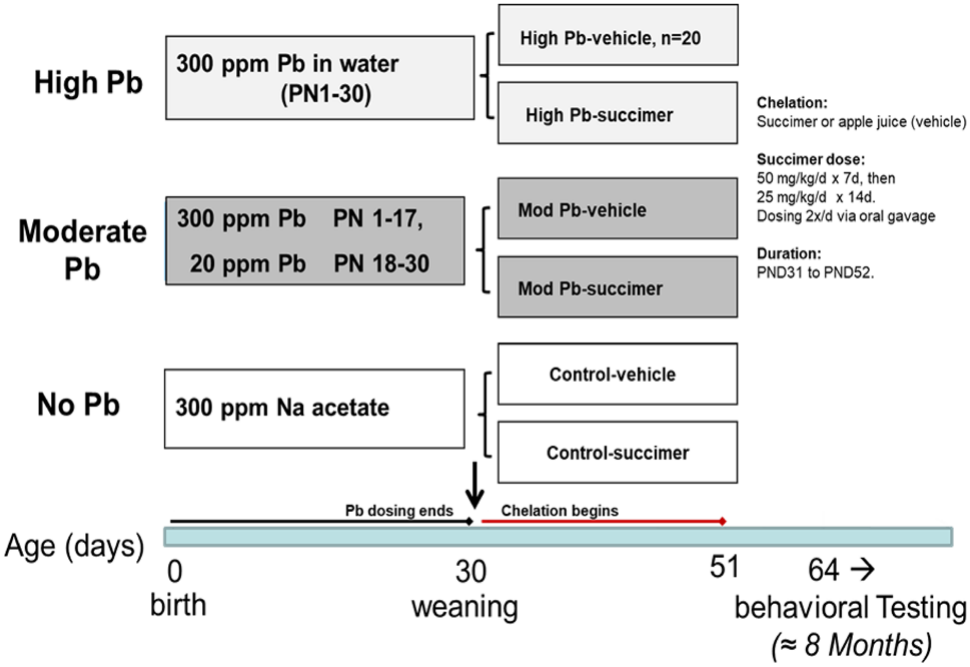
Study design and timing of testing in the rodent succimer chelation of lead (Pb) study (adapted from [16, 167]

The study used a 3 × 2 factorial design, with three levels of lead exposure crossed with two levels of chelation (succimer or vehicle) (see Fig. 1). Rat pups were exposed orally to lead via the lactating dam/drinking water at one of three levels of lead (no lead, moderate lead, high lead) from birth until postnatal day (PND) 30. Both lead groups were asymptomatic and healthy. The PND1–30 period of lead exposure roughly corresponds neurodevelopmentally to the period spanning the third trimester of pregnancy until late childhood/early adolescence in humans. Succimer or apple juice (vehicle) was administered twice daily via oral gavage from PND 31 to PND 52, and behavioral testing began on PND 62. The daily succimer dose was similar to the regimen used clinically [93].

### Behavioral Testing

We describe here a subset of the administered behavioral tests—namely, a series of visual attention tests designed to tap several functions reported to be affected in lead-exposed children, including sustained and selective attention, inhibitory control, learning/associative ability, and arousal regulation. Two of these tasks, the sustained attention and selective attention tasks, are similar to ones commonly used to assess attention in human subjects, such as the Continuous Performance Test and Leonard’s 5-choice Serial Reaction Task [104]. Briefly, in all four of the tasks described here, one of the response cue LEDs above the three response ports of the operant chamber was briefly illuminated on each trial, and the animal was rewarded with a 45-mg food pellet for making a correct nose poke into the port beneath the illuminated LED. This basic rule was mastered during the initial visual discrimination task, and then in the subsequent two attention tasks, the interval between trial onset and cue illumination was increased and the cue duration was shortened (and variable across trials) to place additional demands on focused attention. In the final selective attention task, olfactory distractors were also presented prior to the visual cue on some trials to assess the ability of the animal to maintain attentional focus in the face of potent olfactory distractors. Behavioral testing began on PND 62 and continued 6 days/week for approximately 8 months.

### Pattern of Results

Before detailing specific task results, it may be useful to summarize the overall pattern of effects seen in this study. First, a short period of lead exposure during early development produced lasting cognitive and affective dysfunction, seen months after the cessation of exposure. Second, the 3-week course of succimer chelation produced a significant benefit for the lead-exposed animals, although the degree of benefit varied as a function of both the dose of lead exposure and the specific area of dysfunction. Finally, this same 3-week course of succimer treatment, when administered to animals that were not exposed to lead, produced lasting cognitive and affective dysfunction that was as pervasive and large in magnitude as the dysfunction produced by the higher lead exposure regimen.

### Benefits of Chelation for the Moderate Lead Exposure Group

**Fig. 2:**
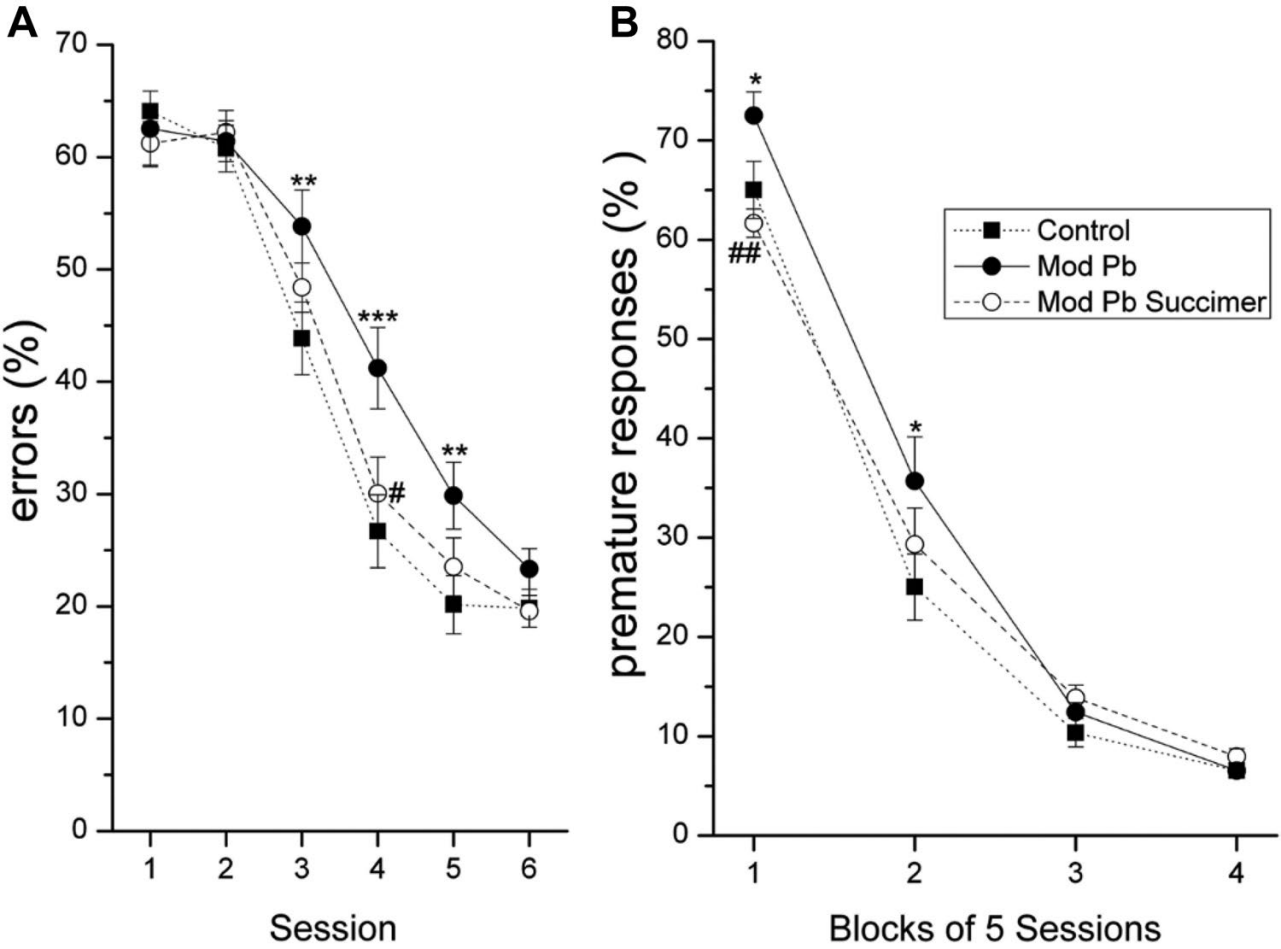
Succimer treatment significantly improved learning ability of the Mod-Pb rats. **A** Visual discrimination task (Mod-Pb-Succ vs. Mod-Pb, main effect contrast, *p* = 0.03). **B** Attention Task 1. Data points are the mean ± SE. *p = 0.056; **p ≤ 0.03, ***p < 0.01, Mod-Pb vs Control; #, *p* = 0.03; # #, *p* = 0.006; Mod-Pb-Succ vs. Mod-Pb*.* Adapted from [16]

We start with findings from the animals in the moderate-lead exposure group (referred to as the Mod-Pb group in the figures). The impairment seen in these animals was very specific and limited to learning ability. As seen in Fig. 2, the rats who received the moderate-lead dose without subsequent chelation learned both the visual discrimination task (panel 2A) and the first focused attention task (panel 2B) more slowly than the controls. In contrast, the rats given this same 30-day lead exposure regimen and treated with succimer chelation performed like the controls (Fig. 2). Thus, succimer chelation fully alleviated the learning deficit in these animals with low/moderate lead exposure.

### Benefits of Chelation for the High-Lead Exposure Group

The results were a bit more complicated for the rats who received the higher lead exposure regimen (referred to as the High-Pb group in the figures). These animals experienced a greater range of cognitive and affective dysfunction than those exposed to the lower lead exposure regimen, and the degree to which succimer was effective for these animals varied across these different functional domains. Several areas of impairment seen in the high-lead group derived little or no benefit from succimer chelation. For example, the impaired learning of the high-lead animals was not alleviated by succimer treatment, nor was their heightened impulsivity (data not shown, [16]).

**Fig. 3:**
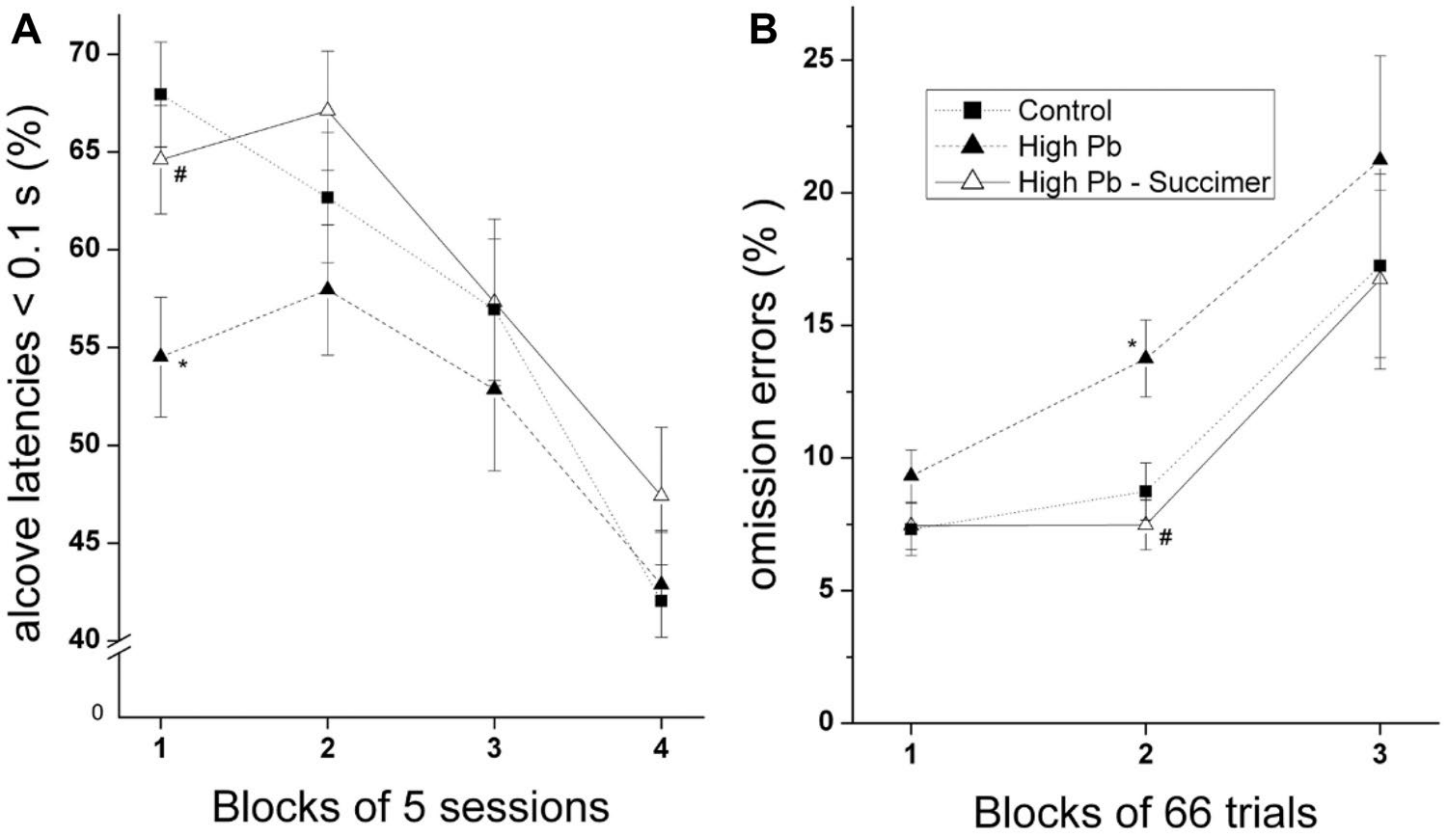
Heightened reactivity to errors of the High-Pb rats was completely normalized by succimer treatment. **A** The percentage of trials in Attention Task 1 for which the latency to enter the testing alcove at trial onset was very short (< 0.1 s), across the 4 blocks of sessions (20 sessions). **B** Percent omission errors for trials following an error in the Sustained Attention Task, plotted as a function of the block of trials within each 200 trial testing session (averaged across the 10 sessions). Data points are means ± SE. *p < 0.01, High-Pb vs. Control. #, *p* < 0.01; High-Pb-Succ vs. High-Pb. Adapted from [16]

In contrast, for other areas of dysfunction in the high-lead animals, succimer chelation was completely effective. One area where succimer was very effective for the high-lead animals was their heightened reaction to committing an error on the previous trial. In this series of tasks, there was evidence that committing an error on the prior trial was disruptive to all groups of animals, including the controls [16]. Specifically, the percentage of all types of errors was significantly higher on trials that followed an error than on trials that followed a correct response. Similarly, the latency to enter the testing alcove and the latency to respond following cue presentation were both longer on trials that followed an error than on trials that followed a correct response [16]. Notably, the performance of the high-lead animals was significantly more disrupted by a prior error than that of the controls, suggesting an impaired ability to regulate the negative arousal produced by committing an error and/or not receiving an expected reward (Fig. 3). This area of dysfunction was totally alleviated by succimer treatment, as seen in Fig. 3. Figure 3B depicts the percentage of omission errors committed across the three blocks of trials in each daily session of the sustained attention task, for trials that followed an error trial. In this task, the pre-cue delay was quite long on some trials to intensify demands on attention. There are several interesting aspects of the findings from this task. First, the percentage of omission errors increases towards the end of each testing session, reflecting the difficulty in sustaining attention across these long sessions (shown in Fig. 3B). Second, the incidence of omission errors is much higher on trials that follow an error than on trials that follow a correct response ([16], data not shown), supporting the inference that committing an error on the prior trial disrupts the ability of the animals to focus attention on the following trial. Moreover, it is striking that the high-lead-exposed animals are impaired, relative to controls, only on trials that follow an error (Fig. 3B). This pattern sheds light on the nature of the impaired performance of the high-lead rats. The fact that the groups did not differ in performance on trials that followed a correct response demonstrates that they understood the rules of the game, that they were as motivated as controls, and that they did not differ from controls in the sensory or motor skills required for performance in this task. The selective impairment seen for trials that followed an error suggests that the non-chelated high-lead animals were less able than controls to regulate the emotional or affective reaction to committing an error, which then manifested as attentional dysfunction. It is notable that the incidence of omission errors for the chelated high-lead group was indistinguishable from the controls (Fig. 3B), indicating that succimer chelation completely alleviated this area of dysfunction. We evaluated error reactivity in several other tasks, and all corroborated this conclusion; it was a very robust and solid finding across tasks.

One possible reason for the finding that succimer treatment was effective in alleviating certain areas of dysfunction but not others may relate to the sensitivity of particular functions to elevated brain lead levels, coupled with the efficacy with which a 3-week course of succimer chelation reduced brain lead. In this study, the fact that the lower lead (Mod-Pb) animals experienced only learning dysfunction, whereas the high-lead animals experienced learning, attentional, and affective dysfunction suggests that learning ability is sensitive to even slightly elevated brain lead levels, contrary to these other areas of functioning. One hypothesis is that the 3-week course of succimer reduced brain lead levels in the moderate-lead group to a level at which functional impairment is not seen [16]. In contrast, this same succimer regimen reduced brain lead of the high-lead group only to the level seen in the non-chelated moderate-lead animals (data not shown, see [16]). It seems likely that a more prolonged succimer regimen or multiple regimens may have further reduced brain lead in the high-lead group and consequently, their dysfunction. In sum, these data demonstrate that under conditions in which succimer significantly reduces brain lead levels, it effectively reduces the cognitive and affective deficits produced by lead exposure.

The benefits of chelation seen in our rodent study were not observed in the TLC study, the one clinical trial of succimer chelation in lead-exposed children that included cognitive outcomes [93, 94]. We propose two possible reasons for the different outcomes. First, it is possible that the succimer treatment protocol used in the TLC trial may not have achieved a sufficient reduction in brain lead levels to improve cognitive functioning. In the TLC study, succimer treatment was discontinued when the blood lead levels of the children reached 15 μg/dL; in light of rodent and primate studies showing that succimer-induced reductions in blood lead greatly overestimate reductions in brain lead levels [97, 102, 103], it is likely that brain lead levels may not have been sufficiently reduced with succimer (versus controls) in the TLC subjects. This suggestion is consistent with the relatively modest 4.5 μg/dL difference in blood lead levels between the succimer- and placebo-treated children in the TLC study over the 6 months following treatment [93]. Second, the disparate outcomes of the TLC trial and the rodent study conducted in our lab may also reflect differences in the nature of the cognitive tasks that were used. In particular, the tasks used here (relative to those in the TLC trial) may have provided more specific indices of the two functional domains most improved by succimer in the present study: associative learning ability and regulation of arousal and/or emotion (indexed by reactivity to errors).

### Effects of Succimer in the Absence of Lead Exposure

One other very interesting and important aspect of the findings from our study pertains to the effects of succimer chelation in the animals that were not exposed to lead. We included this group to gauge the safety of prolonged succimer regimens, which might continue in some cases past the point at which brain lead levels are elevated. Obviously in clinical practice, the physician does not have information about brain lead levels, and we know from our animal model studies that reductions in blood lead levels may not accurately predict reductions in brain lead [97, 102, 103]. In addition, the inclusion of this group was designed to provide information about the safety of succimer in autistic children, due to growing interest in using chelation therapy to treat such children, even in cases where there is no indication of lead exposure.

**Fig. 4:**
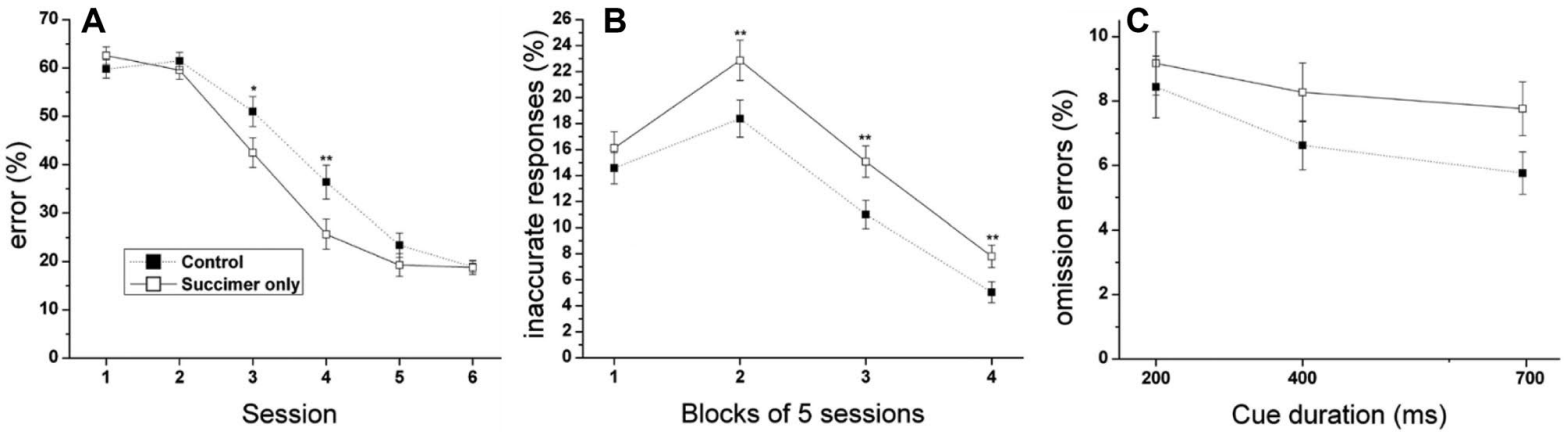
Succimer treatment of the non-lead-exposed rats impaired performance in **A** Visual Discrimination Task (main effect contrast, *p* = 0.04), **B** Attention Task 1, and **C** the Sustained Attention Task (Treatment × Cue Duration: *p* = 0.004). Data points are the mean ± SE. *p = 0.07; **p < 0.05, succimer-only group vs controls. Adapted from [16]

The animals who were chelated but not exposed to lead (called the succimer-only group) learned the initial visual discrimination task more slowly than controls (Fig. 4A). The succimer-only group also performed more poorly than controls in the first attention task, committing a higher percentage of inaccurate responses throughout the 20 sessions on the task (Fig. 4B), indicative of attentional dysfunction. In addition, in the sustained attention task, the succimer-only group also committed a higher incidence of omission errors, particularly for trials with the longer cues (Fig. 4C); this pattern is indicative of lapses in attention, as discussed in Stangle et al. [16]. Moreover, the animals who received succimer in the absence of lead exposure showed additional areas of impairment in the selective attention task relative to controls, with the largest impairment seen for trials that both included a distractor and followed an error on the prior trial (data not shown), indicating both impaired selective attention and an impaired ability to regulate the affective response to committing an error.

### Insights Provided by the Animal Model of Developmental Lead Exposure

Our study provides important insights into the benefits and risks of succimer chelation therapy and also highlights the ways in which animal models can further our understanding of these clinical conditions, in ways that are not possible from studies of humans alone. First, this study provides clear evidence that the administration of succimer, using a regimen sufficient to reduce brain lead levels, can lessen lead-induced impairments in learning ability, attention, and regulation of arousal and/or emotion. These are the first data, to our knowledge, to show that treatment with any chelating agent can alleviate cognitive deficits caused by lead exposure. The primary area of dysfunction seen in the moderate-lead exposure group, impaired learning ability, was completely alleviated by succimer treatment. For the high-lead group, treatment with succimer provided a robust benefit on measures indicative of impaired regulation of arousal or affect—one of the most pervasive areas of impairment seen in this group, and a major cause of their impaired performance overall. However, for the high-lead group, succimer produced only a slight improvement in learning ability and did not lessen the deficient inhibitory control. The high-lead rats treated with succimer performed similarly to non-chelated low/moderate lead-exposed rats — consistent with their comparable brain lead levels—suggesting that a more prolonged succimer treatment may have further improved performance in the high-lead exposed animals.

In addition, the finding that succimer produced lasting adverse effects when administered to non-lead–exposed rats highlights the potential risks of administering succimer or other metal-chelating agents to children who do not have elevated body lead levels. This could be the situation in off-label uses of chelating agents, such as chelation therapy for autistic children who have no history of lead exposure. It is of significant concern that this type of therapy is being advocated as safe and effective for treating autism. It should be noted, however, that as a direct result of the findings from our animal model study, NIH made the decision to permanently halt an ongoing clinical trial of succimer chelation of children with autism (https://www.medpagetoday.com/neurology/autism/10979). This is one of the very few examples where clinical practice was influenced by animal data alone, clearly illustrating the contribution and importance of studies with animal models.

## Public Health Threat to Neurobehavioral Function Posed by Environmental Manganese Exposure

Compared to lead, elevated environmental manganese exposure has only more recently become recognized as a significant public health threat in the US and elsewhere, where vulnerable young children may be exposed to elevated levels of manganese from drinking water [105–108], soil and dust [109–112], and their diet [108, 113–119]. Epidemiological studies have reported associations between environmental manganese exposure and/or exposure biomarkers and deficits in cognition, attention, impulse control/hyperactivity, and psychomotor function in children and adolescents [112, 120–129]. However, unlike lead, there are no well-validated or accepted biomarkers of manganese exposure or health effects, making exposure and exposure-effect assessments in epidemiological studies challenging due to exposure mis-classification, thereby further obscuring the true relationship between environmental manganese exposure and adverse health effects [110, 111, 130–137].

## Environmental Manganese Exposure and ADHD

There is emerging epidemiological evidence that environmental manganese exposure is associated with increased risk of ADHD and/or attention/impulsivity/hyperactivity symptoms in children and adolescents [1, 44, 54, 106, 138–141]. For example, a recent nationwide population-based registry study of over 600,000 children in Denmark reported that exposure to increasing levels of manganese in drinking water was associated with an increased risk of ADHD-Inattentive subtype [141]. After adjusting for covariates, females exposed to high levels of Mn (> 100 μg/L) at least once during their first 5 years of life had a hazard ratio (HR) for ADHD-I of 1.51, while the HR for males was 1.20 when compared with same-sex individuals with peak exposures < 5 μg/L. Broberg et al. [123] investigated if sex and polymorphisms in manganese transporter genes (SLC30A10 and SLC39A8) influenced the association between manganese exposure and ADHD-related behavioral problems in 645 Italian children aged 11–14 years with a wide range of environmental manganese exposure. They reported differences in associations between environmental manganese levels and neurobehavior between sexes, with girls exhibiting higher (worse) self- and parent-reported Conners’ scores in several ADHD-related categories (hyperactivity, inattention, DSM IV Total) at higher environmental manganese levels. For boys, they reported a positive linear relationship with environmental manganese for the parent-reported hyperactivity Conner’s outcome. In earlier studies, Shin et al. [139] evaluated the association of ADHD status with manganese exposure assessed in clinic-referred children with ADHD and control children aged 6–15 years, using hair manganese levels as the exposure biomarker. They found that excess exposure or deficiency of manganese was associated with ADHD among children. Hong et al. [138] evaluated blood manganese concentrations and ADHD diagnosis in a general population of Korean children, aged 8–11 years, using the Child Behavior Checklist (CBCL) instrument. They reported that blood manganese levels were more positively correlated with CBCL scores in ADHD children than in the healthy population. Finally, Oulhote et al. [54] used a questionnaire to assess the association between neurobehavioral functions, including attention, and drinking water manganese exposure, using hair manganese levels as the exposure biomarker in Canadian children, and reported that a 1-SD increase in log_10_ hair manganese levels was associated with a significant difference of − 25% SD in attention. Collectively, these studies support a link between elevated environmental manganese exposure and increased risk of ADHD and/or attention/impulsivity/ hyperactivity symptoms in children and adolescents, although there remains insufficient evidence to support a definite causal relationship [55].

In light of the associations noted above, and their broad implications for human health, we developed a rodent animal model to determine whether developmental environmental manganese exposure can *cause* attention, impulse control, and sensorimotor deficits, and if so, to provide a model to system to elucidate underlying neural mechanisms and test potential therapies. [17, 21, 22, 63, 142–145]. These rodent model studies have employed manganese exposure regimens, sensitive and comprehensive tests of behavioral function, and therapeutic approaches to treat the symptoms that recapitulate conditions in children, so as to maximize human relevance and translational impact.

Specifically, our studies used an oral manganese exposure regimen that corresponds to the exposure risk faced by infants and young children [17, 21, 22, 63, 142–145]. We employed the 5-Choice Serial Reaction Time Task (5-CSRTT) to evaluate visual learning, attention, impulse control, and arousal regulation, because (1) deficits in these functional domains have been reported in environmental epidemiological studies of manganese-exposed children [1, 44, 54, 106, 138–141]; (2) the 5-CSRTT behavioral testing paradigm was developed to mimic neuropsychological tests used in children to evaluate attention and impulse control (e.g., the Continuous Performance Test and Leonard’s 5-choice Serial Reaction Task [104]); and (3) this testing paradigm, when employed with varying trial conditions (e.g., pre-cue delays, presence of olfactory distractors), allows for in-depth assessment of sub-domains of attentional function, in addition to impulse control and arousal regulation. These include attentional preparedness (i.e., ability to orient and attend to the 5-CSRTT response wall in preparation for the salient visual cue), focused attention (ability to maintain attentional focus on the response wall in the face of random prolonged delays between trial onset and presentation of the visual cue), and selective attention (ability to maintain attentional focus on the response wall in the face of olfactory distractors presented immediately prior to the visual cue).

We also utilized a variation of the Montoya Staircase task, modified as described by us and others, as a sensitive test to evaluate forelimb sensorimotor function for manipulating objects [142, 143, 146]. This task was included on the basis of epidemiological studies reporting associations between developmental manganese exposure and impaired psychomotor development, manual dexterity, limb coordination, etc., in children, which in some cases co-occurred with attention and other behavioral deficits [107, 112, 147–150], and because ADHD children are often comorbid for psychomotor disturbances, such as developmental coordination disorder (DCD) [34, 151–155].

**Fig. 5:**
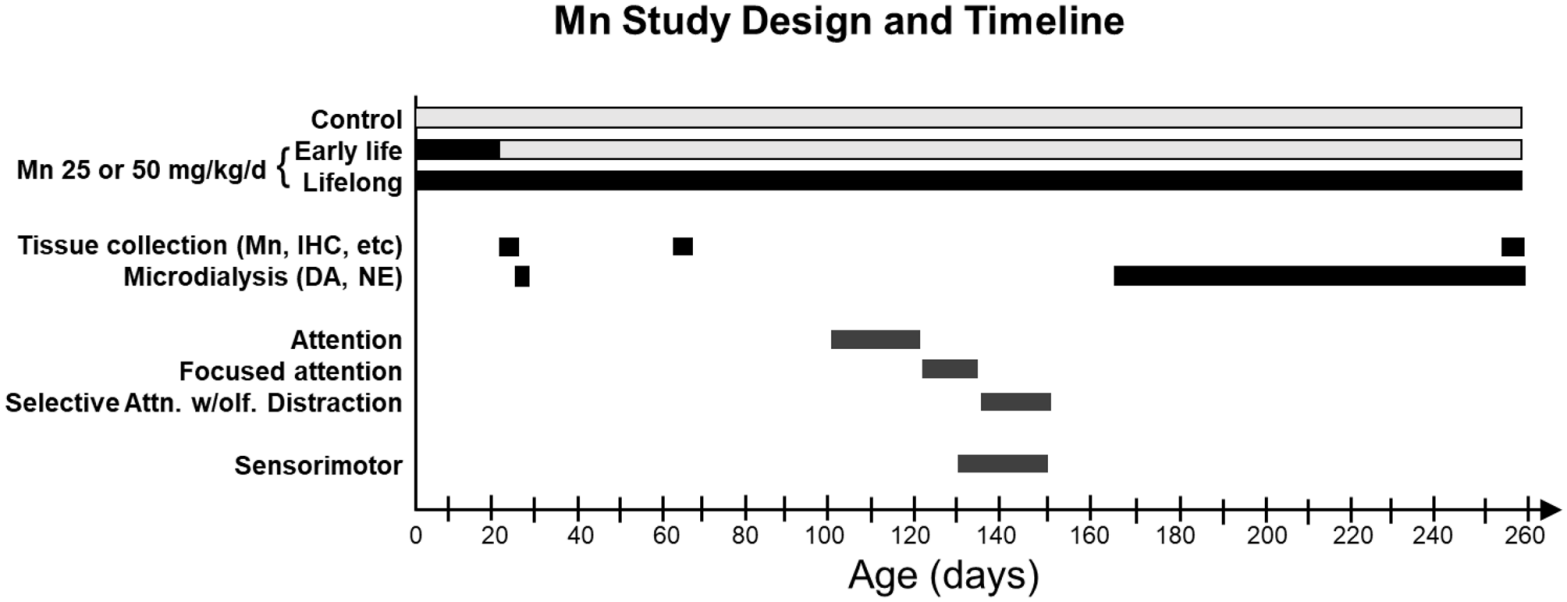
General study design and timing of attention, impulse control, and sensorimotor testing in the rodent studies of developmental (early life) and lifelong oral manganese exposure. Daily oral manganese exposure commenced on postnatal day (PND) 1 and continued until weaning on PND 21 (early life), or continued lifelong via drinking water until the end of the study. The timing of brain and blood tissue collections and microdialysis for evoked release of neurotransmitters [dopamine (DA) and norepinephrine (NE) and metabolites] in littermates of the behaviorally tested animals is also shown. Results from the various outcomes are reported in [17, 22, 143–145]

Below we review and summarize a subset of our animal model findings, since they are among the most conclusive and relevant to human studies of environmental manganese exposure as a causal risk factor for attention deficits in children, and the efficacy of methylphenidate (Ritalin), the most commonly prescribed therapeutic drug for ADHD, to treat those deficits. A general overview of the design and timing of behavioral testing of the manganese rodent studies is shown in Fig. 5.

## Developmental Manganese Exposure Causes Lasting Attention and Sensorimotor Deficits in a Rodent Model of Childhood Manganese Exposure

Our findings have established that developmental manganese exposure can impair attentional preparedness, focused and selective attention, arousal regulation, and sensorimotor function in a rodent model of childhood developmental manganese exposure, and that those deficits last into in adulthood, long after the cessation of exposure at weaning [17, 22, 142, 143, 145]. Several of those findings are worth emphasizing here, including the following: (1) the degree of impairment was most pronounced on 5-CSRTT trial conditions that placed the greatest demands on attentional function; (2) the lasting impairments in attentional function were generally comparable between the manganese groups exposed only during early development (PND 1—21) and groups exposed lifelong through adulthood, indicating that the attentional impairment can be attributed to exposure during that early developmental period; (3) the presence/absence of impulse control deficits due to manganese exposure differed across manganese exposure and animal cohorts/studies; and (4) developmental and lifelong manganese exposure caused selective long-lasting impairment in sensorimotor function in adulthood, although the specific nature of the impairment depended on the dose and duration of exposure.

### Hypofunctioning of the Catecholaminergic System Underlies the Behavioral Deficits Produced by Developmental Manganese Exposure

Our findings (summarized above) led us to hypothesize that developmental manganese exposure disrupts development of catecholaminergic systems in the fronto-cortical-striatal brain areas that modulate attention, impulse control, and sensorimotor functions [156, 157]. In support of this hypothesis, we have shown that oral developmental manganese exposure leads to a hypofunctioning catecholaminergic system in the prefrontal cortex and striatum that persists into adulthood. This includes reduced levels of the key catecholamine synthesis enzyme tyrosine hydroxylase, reduced expression of dopamine and norepinephrine transporters (DAT, NET), altered expression of dopamine receptors DRD1 (reduced) and DRD2 (increased), and lasting reductions in stimulated release of dopamine and norepinephrine [21, 22, 63, 143, 144].

### Therapeutic Approaches for Treating Cognitive and Attentional/Impulse Control Neurobehavioral Symptoms Associated with Environmental Manganese Exposure

In light of the evidence that developmental manganese exposure produced lasting deficits in attentional and sensorimotor function, as well as hypofunctioning of the catecholaminergic fronto-cortico-striatal system, we further hypothesized that methylphenidate (Ritalin) treatment would be effective in alleviating the functional deficits. Methylphenidate is one of the first-line treatments in children and adults with ADHD and related symptoms [1, 44, 158–160]. Preclinical and clinical evidence has shown that therapeutic doses of methylphenidate improve inattention and impulsivity symptoms in humans and animal models of attention-deficit hyperactivity disorder (ADHD) [1, 44, 161, 162], and also ameliorates manual skill deficits in ADHD children with coexisting developmental coordination disorder (ADHD/DCD) [153]. The pharmacologic action of methylphenidate is mediated through its activity as a DAT/NET antagonist, inhibiting the reuptake of synaptic dopamine and norepinephrine, thereby increasing synaptic levels of these neurotransmitters within prefrontal cortical and striatal systems that control/mediate the behavioral and motor functions that are impaired in children with ADHD/DCD. However, alternative mechanisms of methylphenidate efficacy in the treatment of ADHD, involving Wnt- and mTOR-signaling pathways, have also been suggested [159]. Of note here is evidence suggesting that chronic treatment with methylphenidate may alter Wnt and mTOR signaling, and that this action of methylphenidate, perhaps in addition to its acute pharmacologic action to increase synaptic dopamine and norepinephrine, may account for its efficacy to improve attentional function in children [159].

### Oral MPH Treatment Alleviates the Attention, Impulse Control, and Sensorimotor Deficits Caused by Developmental Manganese Exposure

**Fig. 6:**
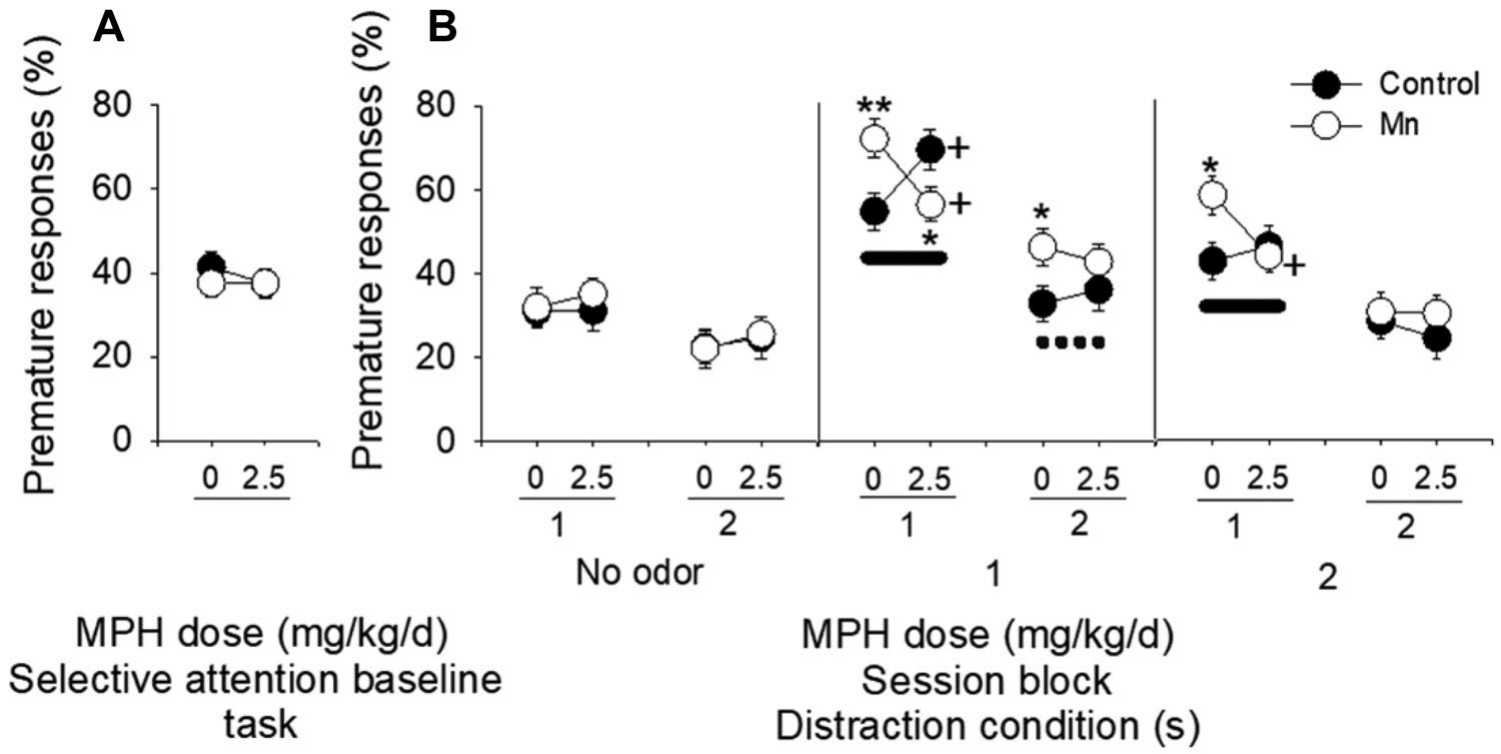
Mn exposure increased distractor-induced impulsivity in the selective attention task, and MPH treatment alleviated the Mn effect while increasing impulsivity in rats never exposed to Mn. Mean percent premature responses (± SE) for the control and Mn-exposed groups in **A** the baseline attention task, as a function of MPH dose, and in **B** the selective attention task, as a function of MPH dose, session block (three test session days/block), and distractor condition (*n* = 10/group). * and ** indicate significant differences between the Mn and control groups at *p* ≤ 0.05 or *p* ≤ 0.01, respectively, for each of the 0 or 2.5 mg MPH/kg/d treatment conditions. + indicates significant differences between the MPH and vehicle-treated groups at *p* ≤ 0.05 for each of the control or the Mn-exposed conditions. The full line in **B** indicates *no* difference between the Mn + MPH group and the Control + Veh groups, reflecting the therapeutic effect of MPH treatment in Mn-exposed rats. The dotted line in **B** indicates a significant difference between the Mn + MPH and the Control + Veh groups at *p* ≤ 0.05, reflecting the absence of a therapeutic MPH effect on Mn-exposed rats. Adapted from [145]

**Fig. 7:**
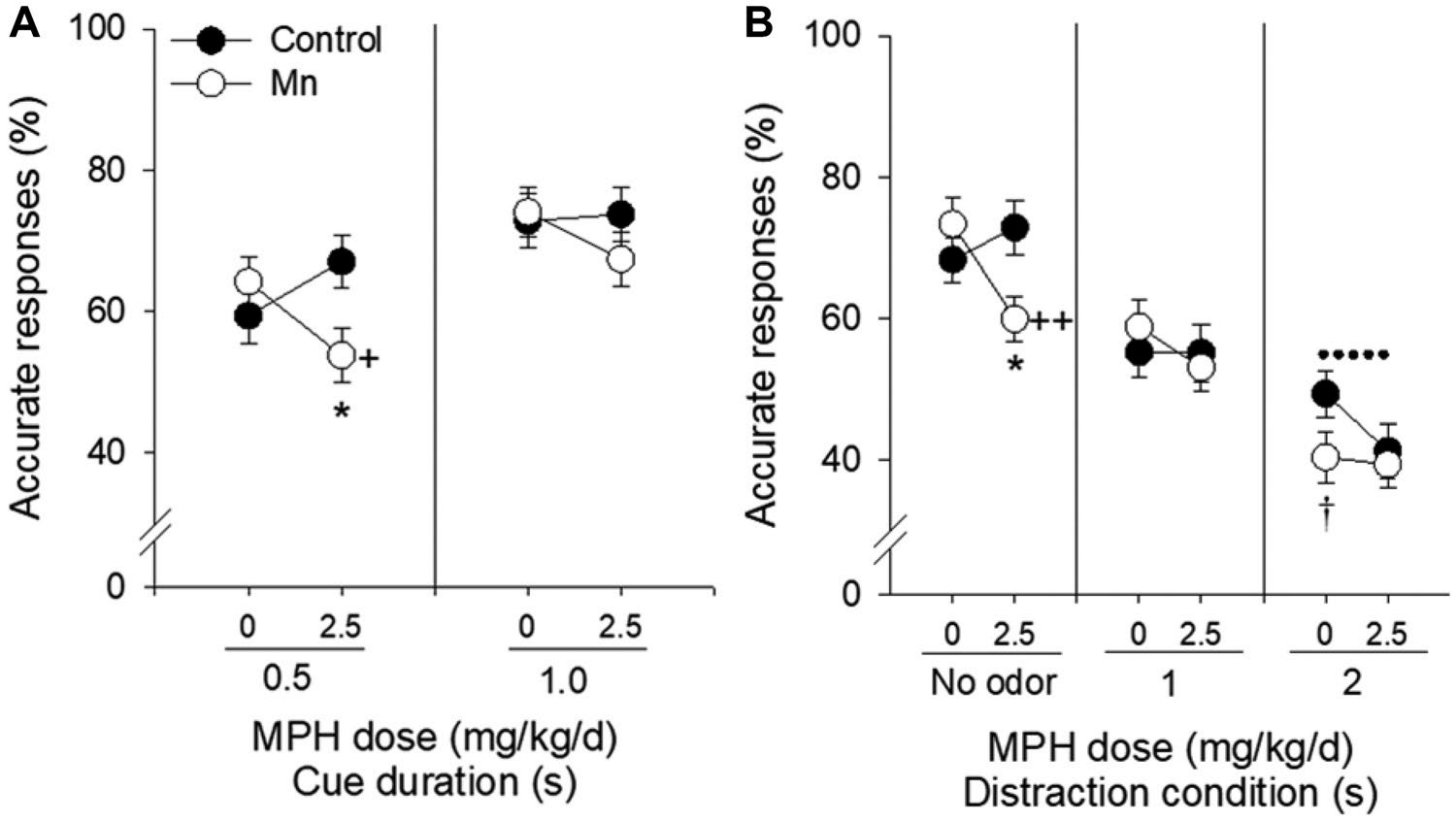
MPH did not alleviate the Mn-induced impairment in selective attention and impaired focused attention in the Mn rats. Mean percent accurate responses (± SE) for the control and Mn-exposed groups in **A** the selective attention baseline attention task, as function of MPH dose and duration of the visual cue, and in **B** the selective attention task, as function of MPH dose and distractor condition (*n* = 10/group). † indicates a trending significant difference between the Mn + Veh and Control + Veh groups at 0.05 < *p* ≤ 0.10. + and + + indicate significant differences between the Mn + MPH and Mn + Veh groups at *p* ≤ 0.05 and *p* ≤ 0.01 in (**A** and **B**), respectively. * indicates a significant difference between the Mn + MPH and control + MPH groups at *p* ≤ 0.05. The dotted line in **B** indicates a significant difference between the Mn + MPH and the control + Veh groups at *p* ≤ 0.05, reflecting the absence of a therapeutic effect of MPH on Mn-exposed rats. Adapted from [145]

**Fig. 8:**
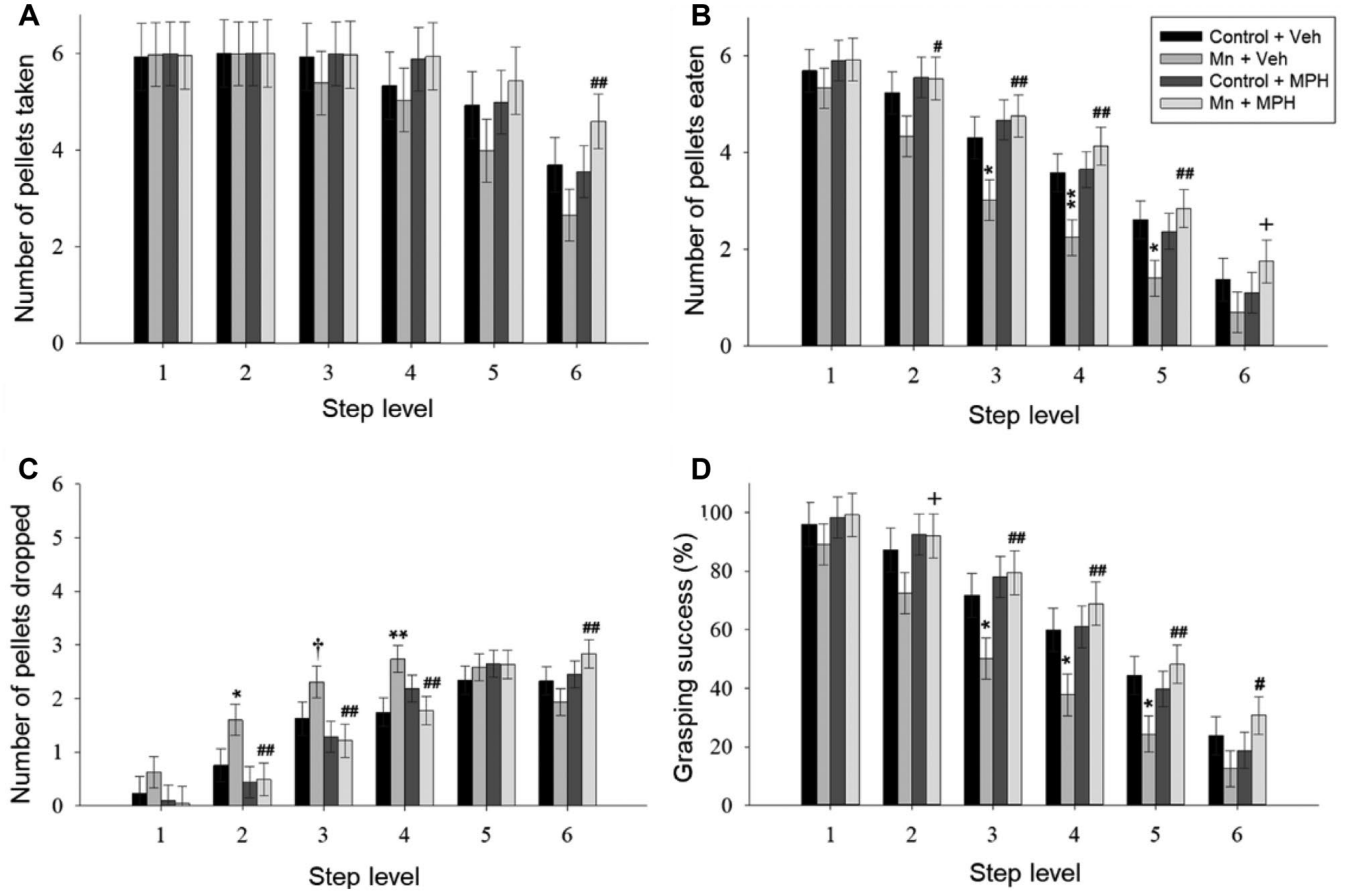
Chronic postnatal Mn exposure reduced the number of pellets eaten from the more difficult to reach lower steps of the staircase, and MPH treatment fully normalized this deficit. **A** Number of pellets taken, **B** number of pellets eaten, **C** number of pellets dropped, and **D** percentage of grasping success by control and Mn-exposed groups not treated with MPH or treated with MPH, as a function of step level. Bars represent the mean ± SEM (*n* = 10/group). * and ** indicate *p* ≤ 0.05 and *p* ≤ 0.01, respectively, versus Control + VEH group; # and ## indicate *p* ≤ 0.05 and *p* ≤ 0.01, respectively, versus Mn + VEH group; † indicates 0.05 < *p* ≤ 0.10 versus control + VEH group; + indicates 0.05 < *p* ≤ 0.10 versus Mn + VEH group. Adapted from [143]

To test the efficacy of methylphenidate for ameliorating the manganese-induced attentional and motor dysfunction, we conducted studies in which animals were exposed to manganese developmentally and throughout life (50 mg/kg/d, as noted above), and treated daily with oral methylphenidate (2.5 mg/kg/d) over the 3 days of the selective attention baseline task (no olfactory distractors) followed by 12 days of testing on the selective attention task and the Montoya staircase sensorimotor task [143, 145]. This single methylphenidate dose was selected for our first study, as it is at the higher range of doses used clinically. To assess methylphenidate efficacy, we first again established that developmental manganese exposure caused behavioral deficits consistent with our prior studies [17, 21, 142]. For example, we found that lifelong postnatal manganese exposure increased distractor-induced impulsivity (Fig. 6B), impaired selective attention (Fig. 7B), and caused deficits in sensorimotor function in adulthood (Fig. 8) [143, 145], generally consistent with our prior studies.

The efficacy of this oral dose of methylphenidate varied for the different areas of dysfunction. Importantly, we found that this therapeutically relevant oral methylphenidate regimen alleviated the impulse control deficits caused by manganese exposure (Fig. 6; [145]). Specifically, the manganese group treated with methylphenidate committed significantly *fewer* premature responses than the manganese-vehicle group for the 1 s and 2 s distraction condition trials during session block 1 of the selective attention task, performing at a level that was not different from the Control + Veh group (Fig. 6B). In contrast, this single daily oral dose of methylphenidate was not effective in ameliorating the deficit in selective attention in the manganese-exposed rats and actually impaired the focused attention ability of these animals (Fig. 7A, B; [145]). Specifically, the manganese-exposed animals were more distracted by the olfactory distractors than controls, an area of dysfunction that was not improved by methylphenidate treatment at this dose (Fig. 7B). Moreover, for the trials without distractors, response accuracy of the manganese animals treated with methylphenidate was even lower than for the manganese animals treated with vehicle (Fig. 7B). Methylphenidate also further reduced response accuracy of the manganese animals for trials in the baseline selective attention task (no distractors) with the briefest visual cue (Fig. 7A).

However, it is important to note that in more recent studies evaluating a range of oral methylphenidate doses over several successive attention tasks lasting ~ 30 days, we found that a lower dose of methylphenidate (0.5 mg/kg/d) was extremely effective in alleviating the attentional dysfunction produced by developmental manganese exposure. Specifically, in a study including four different oral methylphenidate doses (0, 0.5, 1.5, or 3 mg/kg/d), we observed an inverted U-shaped dose–response for attentional accuracy in the manganese-exposed animals, but not the controls, with the lowest 0.5 mg/kg dose fully ameliorating the manganese attentional deficits, and again no benefit or adverse effects seen at the higher doses (data not shown; [163]). Interestingly this pattern was not seen for controls, consistent with our evidence that developmental manganese exposure produces lasting changes in the catecholaminergic system.

We also found that oral treatment with methylphenidate (2.5 mg/kg/d) fully alleviated the manganese-induced impairment in sensorimotor function in adult rats, but did not alter sensorimotor performance in control animals (Fig. 8) [143]. For example, the manganese-exposed group treated with methylphenidate performed similarly to controls (with or without MPH) and took and ate significantly more pellets than their manganese + vehicle counterparts at staircase step levels 2–6 (Fig. 8A, B), and also dropped significantly fewer pellets than the manganese + vehicle group from step levels 2–4 (Fig. 8C). As a result, the percent grasping success of the manganese + methylphenidate group was significantly greater than that of the manganese + vehicle group at step levels 2–6 (Fig. 8D).

### Summary of Insights Provided by Our Studies of Manganese Exposure and ADHD-Like Symptoms

Our animal model of developmental manganese exposure has provided a number of important contributions to our understanding of the role of environmental exposures as contributors to ADHD and attentional/sensorimotor deficits more generally. First, they demonstrate that developmental manganese exposure, at levels of direct relevance to infants and young children, can cause lasting attentional and sensorimotor deficits. Moreover, they provide important insight into the specific nature of the lasting attention and sensorimotor deficits, and how they vary with the dose and timing of exposure, which helps explain the variation in findings reported in the epidemiological literature regarding the neurobehavioral effects of childhood manganese exposure [112, 120–129]. Second, our studies have shown that the lasting attentional and sensorimotor deficits are accompanied by lasting hypofunctioning of the catecholaminergic system in the prefrontal cortex and striatum (i.e., the fronto-cortico-striatal system), including reduced stimulated release of dopamine and norepinephrine, and altered expression of key catecholaminergic system proteins. These findings are consistent with neuropsychological and imaging studies in children that have shown that ADHD (and attentional dysfunction more broadly) is generally associated with hypo-functioning of catecholaminergic systems within the cortico-striatal loop [1, 164–166]. Finally, our studies have demonstrated that oral methylphenidate, the most commonly prescribed medication for the treatment of ADHD symptoms, was fully efficacious in alleviating the attentional, impulse control, and sensorimotor deficits, albeit at a lower oral methylphenidate dose for the attentional deficits (0.5 mg/kg/d) compared to the sensorimotor deficits (2.5 mg/kg/d).

## Conclusions

Animal models play several key roles in studying links between neurotoxicant exposure and childhood behavioral disorders such as ADHD. First, they are critical for establishing causal relationships between exposure to a particular neurotoxicant and dysfunction in various cognitive and affective domains. Establishing definitive causal links is not possible in human epidemiological studies due to the challenges associated with exposure assessment, and in fully controlling for the multitude of sociodemographic risk factors, such as poverty, that are often correlated with neurotoxicant exposure. Second, following the successful development of an animal model which recapitulates the key phenotypic features of the behavioral disorder, such models can be used to elucidate the underlying neural mechanisms and test the efficacy of potential therapies.

Our animal model studies of early developmental exposure to lead and manganese illustrate these contributions. Results from our animal model of developmental lead exposure provided the first evidence that chelation therapy can alleviate certain types of lead-induced cognitive dysfunction, which is an important contribution since the neurotoxic effects of early lead exposure are considered to be irreversible. Moreover, because we were able to measure the effects of the chelator on both cognitive functioning and brain lead levels, we were able to shed light on one possible reason for the disparate conclusions reached about the effectiveness of succimer chelation in the human and animal studies, i.e., in our rodent model study, chelation was effective in lessening the adverse cognitive effects of lead exposure, whereas the human clinical trial of this treatment found no benefit. Specifically, in our studies, cognitive benefits were seen with chelation, but only under conditions (lead exposure and chelation dose) where a substantial reduction in brain lead was achieved. The human clinical trial most likely did not achieve a sufficient reduction in brain lead to see a cognitive benefit, based on the small changes in blood lead that were observed. Finally, the finding that succimer produced lasting adverse effects when administered to non-lead–exposed rats highlights the potential risks of administering succimer or other metal chelating agents to children who do not have elevated tissue lead levels. This situation could occur with prolonged chelation of lead-exposed children or with the use of succimer in children (e.g., with autism spectrum disorder) without a history of lead exposure. It remains of significant concern that this type of therapy is being advocated as safe and effective for treating autism.

Similarly, our animal model of developmental manganese exposure has made a number of important contributions to understanding of the role of environmental exposures as contributors to ADHD and attentional/sensorimotor deficits more generally. First, they demonstrate that developmental manganese exposure, at levels of direct relevance to infants and young children, can cause lasting attentional and sensorimotor deficits. Second, our studies have shown that the lasting attentional and sensorimotor deficits are accompanied by lasting hypofunctioning of the catecholaminergic system in the prefrontal cortex and striatum. Finally, our studies have demonstrated that oral methylphenidate, the most commonly prescribed medication for the treatment of ADHD symptoms, was fully efficacious in alleviating the attentional, impulse control, and sensorimotor deficits, albeit at a lower oral methylphenidate dose for the attentional deficits (0.5 mg/kg/d) compared to the sensorimotor deficits (2.5 mg/kg/d).

In sum, findings from the studies reviewed here have important implications for the use of chelation therapy in treating children exposed to lead, and for the use of methylphenidate (or other catecholaminergic agonists) to treat the behavioral disorders associated with elevated manganese exposure in humans. In addition, these animal models, including the exposure parameters and behavioral tests, can be valuable for testing future therapies as they become available. Future work should continue to focus on the development and use of animal models that appropriately recapitulate the complex behavioral phenotypes of behavioral disorders to (a) determine the mechanistic basis of the behavioral deficits caused by developmental exposure to environmental toxicants and (b) elucidate the molecular basis of existing and newly developed therapies.

## Supplementary Information

Below is the link to the electronic supplementary material.

Supplementary file1 (PDF 507 kb)

Supplementary file2 (PDF 498 kb)

## Data Availability

The authors confirm that the data supporting the findings of the studies reffered to in the graphical figures are available within the original cited articles, their supplementary materials, and/or via the corresponding authors.
